# Phenotyping congenital anomalies in England and Scotland: a comparison of three coding clusters using retrospective hospital data

**DOI:** 10.23889/ijpds.v1i1.372

**Published:** 2017-04-19

**Authors:** Linda Wijlaars, Ania Zylbersztejn, Maximiliane Verfürden, Ruth Gilbert, Pia Hardelid

**Affiliations:** 1 University College London Institute of Child Health; 2 University College London

## Objectives

We aimed to compare prevalence rates of complex congenital anomalies (CCAs) in the first two years of life in England and Scotland using two published CCA diagnosis code lists (phenotypes) developed to identify children who require high levels of healthcare.

## Approach

We developed birth cohorts of singleton, hospital live births between 1998-2011 using birth registration, hospitalisation and mortality register datasets from England (n=8,157,213) and from Scotland (n=731,377) from. Children were followed up until 2 years of age.

We compared prevalence rates of congenital anomalies using two code lists developed to identify CCAs: a UK chronic condition code list developed by Hardelid et al. and a US medical complexity code list developed by Feudtner et al. Children were classified as having a CCA if a diagnosis code was included in any admission during follow-up as a primary or subsidiary diagnosis.

To assess the level of healthcare attention required, we compared the proportion of children who had at least one re-admission to hospital in the first two years of life.

## Results

Rates of CCAs in England and Scotland were higher using the Hardelid phenotype (2.8% for both countries) than when using the Feudtner phenotype (1.8% in England and 1.5% in Scotland). Rates of CCAs increased in both England and Scotland between 1998 and 2011. The increase was more notable using the Hardelid phenotype (37.2% and 20.2%, in England and Scotland, respectively) compared to the Feudtner phenotype (35.6% and 13.0%). Prevalence rates using the Hardelid phenotype were similar to EUROCAT figures.

The proportion of children with at least one re-admission to hospital was higher in the Feudtner code list (69.2% in England and 84.8% in Scotland), than for Hardelid’s code list (67.9% and 72.0% in England and Scotland, respectively). The proportions of children with any of the CCA phenotypes were higher than compared to children without CCAs (24.4% in England and 25.8% in Scotland).

## Conclusion

Prevalence of CCAs and associated hospital use vary depending on choice of CCA code list. Our findings suggest that Feudtner's code list identifies children with higher level of healthcare needs. The impact of coding methods for defining CCAs in administrative hospital records should be explored and externally validated when comparing prevalence of CCAs and associated hospital use between countries.

The impact of coding method for defining CCAs in administrative hospital records should be explored and externally validated when comparing prevalence and admission rates between countries.

